# Supplementary material for: Research of the impact of economic decline on air quality in Wuhan under COVID-19 epidemic
Source: PLoS One. 2023 Mar 9;18(3):e0282706. doi: 10.1371/journal.pone.0282706 (PMC9997873; doi:10.1371/journal.pone.0282706)
Supplement: S1 Appendix — (DOCX) [file pone.0282706.s001.docx]

# The appendix

**PGSA Algorithm steps and principles**.

The implementation steps of the PGSA are as follows：

***Step* 1** Determine the initial growth point$x^{0}\in x$and the step size $l/1000$, in which $l$ is the length of the bounded closed box. Let$X_{\min}=x^{0}$, $F_{\min}=f(x^{0})$ , in which$f(x^{0})$ is the backlight function of $x^{0}$.

***Step* 2** Let $x^{0}$ be the center point, and draw a line parallel to the $x$-axis and another parallel to the$y$-axis along a two-dimensional plane; $a_{1}\leq x_{1}^{0}\leq b_{1},a_{2}\leq x_{2}^{0}\leq b_{2},...,a_{t}\leq x_{t}^{0}\leq b_{t}$ is then derived as new branches of growth. Look for $S_{i_{1}j_{1}}^{0}(1\leq i_{1}\leq t,1\leq j_{1}\leq k_{1})$ in $E$, in which $S_{i_{1}j_{1}}^{0}$ is the $j_{1}$th growth point on the $i_{1}$th branch.

***Step* 3** Compare $f(S_{i_{1}j_{1}}^{0})$ with $F_{\min}$, if $f(S_{i_{1}j_{1}}^{0})\leq F_{\min}$, then $X_{\min}=S_{i_{1}j_{1}}^{0},F_{\min}=f(S_{i_{1}j_{1}}^{0})$. Otherwise, keep $X_{\min}$and $F_{\min}$ unchanged.

***Step* 4** If$f(x^{0})\leq f(S_{i_{1}j_{1}}^{0})$, then its auxin concentration is $C_{S_{i_{1}j_{1}}^{0}}=0$; otherwise, solve $C_{S_{i_{1}j_{1}}^{0}}$as follows:

$C_{S_{i_{1}j_{1}}^{0}}=\frac{f(x^{0})-f(S_{i_{1}j_{1}}^{0})}{\sum_{i_{1}=1}^{m} \sum_{j_{1}=1}^{k_{1}} [f(x^{0})-f(S_{i_{1}j_{1}}^{0})]}$ （1）

***Step* 5** Using the concentration of auxin for all growth points to establish a concentration point between 0 and 1, let $\zeta_{0}$ be a random point for the interval number, and thus$\sum_{i_{1}=1}^{r_{1}} \sum_{j_{1}=1}^{t_{1}-1} C_{S_{i_{1}j_{1}}^{0}}<\zeta_{0}<\sum_{i_{1}=1}^{r_{1}} \sum_{j_{1}=1}^{t_{1}} C_{S_{i_{1}j_{1}}^{0}}$. Next, choose$S_{r_{1}t_{1}}^{0}$ as the new growth point; meanwhile, $x^{1}=S_{r_{1}t_{1}}^{0}\text{，}X_{\min}=x^{1},F_{\min}=f(x^{1})$.

***Step* 6** Let $x^{1}$ be the ideal point, and draw a line parallel to the $x$-axis and another parallel to the $y$-axis along a two-dimensional plane; $S_{i_{1}j_{1}}^{1}(1\leq i_{2}\leq t,1\leq j_{2}\leq k_{1})$is then derived as a new branch of growth. Afterward, search $S_{i_{1}j_{1}}^{1}(1\leq i_{2}\leq t,1\leq j_{2}\leq k_{1})$ in $E$.

***Step* 7** Comparing$f(S_{i_{2}j_{2}}^{0})$ with $F_{\min}$, if$f(S_{i_{2}j_{2}}^{0})\leq F_{\min}$, then$X_{\min}=S_{i_{2}j_{2}}^{0},F_{\min}=f(S_{i_{2}j_{2}}^{0})$; otherwise, keep $X_{\min}$and$F_{\min}$unchanged.

***Step* 8** Solve $C_{S_{i_{1}j_{1}}^{0}}$and$C_{S_{i_{12}j_{2}}^{1}}$, and if$f(x^{0})\leq f(S_{i_{1}j_{1}}^{1})$, then its auxin concentration is $C_{S_{i_{1}j_{1}}^{0}}=0$; otherwise, solve $C_{S_{i_{1}j_{1}}^{0}}$ as follows:

$C_{S_{i_{1}j_{1}}^{0}}=\frac{f(x^{0})-f(S_{i_{1}j_{1}}^{0})}{\sum_{i_{1}=1}^{m} \sum_{j_{1}=1}^{k_{1}} [f(x^{0})-f(S_{i_{1}j_{1}}^{0})]+\sum_{i_{1}=1}^{m} \sum_{j_{1}=1}^{k_{2}} [f(x^{0})-f(S_{i_{2}j_{2}}^{1})]}$ (2)

$C_{S_{i_{2}j_{2}}^{1}}=\frac{f(x^{0})-f(S_{i_{2}j_{2}}^{1})}{\sum_{i_{1}=1}^{m} \sum_{j_{1}=1}^{k_{1}} [f(x^{0})-f(S_{i_{1}j_{1}}^{0})]+\sum_{i_{1}=1}^{m} \sum_{j_{1}=1}^{k_{2}} [f(x^{0})-f(S_{i_{2}j_{2}}^{1})]}$ (3)

***Step* 9** Using the concentration of auxin for all growth points to establish a concentration point between 0 and 1, let $\zeta_{1}$ be another random point for the interval number, and thus $\sum_{i_{1}=1}^{r_{2}} \sum_{j_{1}=1}^{t_{2}-1} C_{S_{i_{1}j_{1}}^{0}}<\zeta_{1}\leq\sum_{i_{1}=1}^{r_{2}} \sum_{j_{1}=1}^{t_{2}} C_{S_{i_{1}j_{1}}^{0}}$.Next, choose$S_{r_{1}t_{1}}^{0}$as the new growth point, and meanwhile$x^{2}=S_{r_{1}t_{1}}^{0}\text{，}X_{\min}=x^{2},F_{\min}=f(x^{2})$; otherwise, $\sum_{i_{1}=1}^{m} \sum_{j_{1}=1}^{k_{1}} C_{S_{i_{1}j_{1}}^{0}}+\sum_{i_{1}=1}^{r_{2}} \sum_{j_{1}=1}^{t_{2}} C_{S_{i_{2}j_{2}}^{1}}<\zeta_{1}\leq\sum_{i_{1}=1}^{m} \sum_{j_{1}=1}^{k_{1}} C_{S_{i_{1}j_{1}}^{0}}+\sum_{i_{1}=1}^{r_{2}} \sum_{j_{1}=1}^{t_{2}} C_{S_{i_{2}j_{2}}^{1}}$, and then choose $S_{r_{2}t_{2}}^{1}$as the new growth point, through which we suppose $x^{2}=S_{r_{1}t_{1}}^{1}\text{，}X_{\min}=x^{2},F_{\min}=f(x^{2})$.

***Step* 10**  Repeat steps 6 through 9 until $F_{\min}$ remains unchanged, and then determine whether $x^{*}=X_{\min}$ is the global optimal solution, and if so, the iterations end.
