# Supplementary material for: Research of the impact of economic decline on air quality in Wuhan under COVID-19 epidemic
Source: PLoS One. 2023 Mar 9;18(3):e0282706. doi: 10.1371/journal.pone.0282706 (PMC9997873; doi:10.1371/journal.pone.0282706)
Supplement: Attachment — Submitted filename: renamed_9a27e.docx [file pone.0282706.s003.docx]

Reviewer #1:

**1. Response to comment:** The authors have improved their manuscript. However, I recommend citing more articles on the Covid pandemic, such as：

https://doi.org/10.1007/s41651-020-00064-5

https://doi.org/10.1016/j.atmosres.2020.105362

https://doi.org/10.3390/rs12101576

https://doi.org/10.3390/rs12101613

https://doi.org/10.4209/aaqr.2020.05.0226

**Response:**

Thank you very much for your suggestion. We have carefully studied these references and cited them as references for the paper ([6], [7], [10], [32], and [33]).

Journal Requirements:

Please review your reference list to ensure that it is complete and correct. If you have cited papers that have been retracted, please include the rationale for doing so in the manuscript text, or remove these references and replace them with relevant current references. Any changes to the reference list should be mentioned in the rebuttal letter that accompanies your revised manuscript. If you need to cite a retracted article, indicate the article’s retracted status in the References list and also include a citation and full reference for the retraction notice.

**Response:**

Thank you very much for your suggestion. We checked the list of reference list to ensure its validity, and updated some references according to the suggestions of Reviewer #1.
